# Detecting Key Genes Regulated by miRNAs in Dysfunctional Crosstalk Pathway of Myasthenia Gravis

**DOI:** 10.1155/2015/724715

**Published:** 2015-02-01

**Authors:** Yuze Cao, Jianjian Wang, Huixue Zhang, Qinghua Tian, Lixia Chen, Shangwei Ning, Peifang Liu, Xuesong Sun, Xiaoyu Lu, Chang Song, Shuai Zhang, Bo Xiao, Lihua Wang

**Affiliations:** ^1^Department of Neurology, The Second Affiliated Hospital, Harbin Medical University, Harbin, Heilongjiang 150081, China; ^2^Department of Neurology, Xiangya Hospital, Central South University, Changsha, Hunan 410008, China; ^3^College of Bioinformatics Science and Technology, Harbin Medical University, Harbin, Heilongjiang 150081, China

## Abstract

Myasthenia gravis (MG) is a neuromuscular autoimmune disorder resulting from autoantibodies attacking components of the neuromuscular junction. Recent studies have implicated the aberrant expression of microRNAs (miRNAs) in the pathogenesis of MG; however, the underlying mechanisms remain largely unknown. This study aimed to identify key genes regulated by miRNAs in MG. Six dysregulated pathways were identified through differentially expressed miRNAs and mRNAs in MG, and significant crosstalk was detected between five of these. Notably, crosstalk between the “*synaptic long-term potentiation*” pathway and four others was mediated by five genes involved in the MAPK signaling pathway. Furthermore, 14 key genes regulated by miRNAs were detected, of which six—*MAPK1, RAF1, PGF, PDGFRA, EP300, and PPP1CC*—mediated interactions between the dysregulated pathways. *MAPK1* and *RAF1* were responsible for most of this crosstalk (80%), likely reflecting their central roles in MG pathogenesis. In addition, most key genes were enriched in immune-related local areas that were strongly disordered in MG. These results provide new insight into the pathogenesis of MG and offer new potential targets for therapeutic intervention.

## 1. Introduction

Myasthenia gravis (MG) is a neuromuscular autoimmune disorder caused by antibodies that attack the acetylcholine receptor (AChR), leading to muscle fatigue and weakness. The pathogenesis of MG is not fully understood but involves the interaction of genetic, environmental, and immunological factors, thymic abnormalities, and age [1]. Despite advances in treatments, there are none that specifically target the autoimmune deficiency in MG. Several recent studies have shown that various microRNAs (miRNAs) are aberrantly expressed in MG, presenting new possibilities for understanding disease pathogenesis as well as for diagnosis and treatment.

miRNAs are small (~22 nucleotide) noncoding RNA molecules that regulate the expression of their target mRNAs at the posttranscriptional level. miRNAs regulate a wide range of biological processes, including development, cell differentiation and proliferation, metabolism, and apoptosis [2, 3], and contribute to the pathogenesis of a variety of autoimmune disorders including MG [4]. For example, miR-320a and let-7c are downregulated in MG patients relative to healthy control subjects [5, 6], and miRNA-146a is upregulated in MG patients and was found to act on B cells expressing AChR, thereby contributing to the development of MG [7]. A recent study showed that serum levels of a set of miRNAs were reduced in MG patients, and some were differentially expressed in early and late onset MG [8]. However, it remains unclear how the dysregulation of miRNAs leads to MG.

Most association studies aiming to identify candidate genes in MG have focused on a specific gene or several independent genes [9–12]. However, there is increasing evidence to suggest that MG arises from the interaction of multiple genes. For instance, several members of the nuclear factor- (NF-) *κ*B signaling pathway work cooperatively to trigger an immune response in MG [13], and a recent study found that miR-155 was upregulated in MG patients, while suppressing miR-155 impaired NF-*κ*B signaling [14]. Since functionally connected pathways crosstalk through common sets of genes [15], identifying miRNAs and their target genes that are dysregulated in MG is critical for developing effective treatment strategies.

In this study, pathways that are dysregulated in MG were identified by pathway enrichment analysis. Some MG-related genes were found to mediate crosstalk between these pathways. Key genes regulated by miRNAs were identified by combining computational predictions with mRNA expression profiles, and these were found to mediate crosstalk and were enriched in disordered local areas of pathways (LAPs) related to MG. The findings reveal a set of genes that play key roles in the pathogenesis of MG and can potentially serve as novel therapeutic targets.

## 2. Materials and Methods

### 2.1. Data Sources

#### 2.1.1. Expression Profiles of mRNA and miRNA

The mRNA expression profiles for MG were obtained from ArrayExpress database (accession number E-MEXP-518, Raw data in Optional Supplementary Material, available online at http://dx.doi.org/10.1155/2014/724715) and comprised 30 samples from 20 MG patients and 10 control subjects, for a total of 12,814 genes [16]. The miRNA expression profiles have been previously published and were from three MG patients and three matched healthy controls [5], for a total of 866 mature human miRNAs.

#### 2.1.2. miRNA Target Genes

miRNA target genes were determined using the following miRNA target prediction tools: miRanda (August 2010 release) [17], TargetScan (release 6.2) [18], DIANA-microT (version 5.0) [19], mirSVR [20], PicTar (five-way) [21], RNA22 [22], RNAhybrid [23], PITA (version 6) [24], MirTarget2 (version 4.0) [25], and TargetMiner [26]. To minimize the false positives, only genes predicted by at least four tools were selected as putative miRNA targets for pathway analysis.

### 2.2. Methods

#### 2.2.1. Identification of Differentially Expressed mRNAs and miRNAs

Raw data from mRNA expression profiles were quantified using the global locally weighted scatterplot smoothing (Lowess) transformation and were transformed in log2-scale; differentially expressed mRNAs were identified by significance analysis of microarray data. A false discovery rate (FDR) < 0.05 and fold change (FC) > 2 were considered statistically significant. Background subtraction and normalization were performed for miRNA expression profiles and FC > 2 were selected as differentially expressed miRNAs.

#### 2.2.2. Pathway Enrichment and Crosstalk Analysis

The Database for Annotation, Visualization, and Integrated Discovery was used for the KEGG pathway enrichment analysis [27].* P* values were calculated with Fisher's exact test. A cutoff value of *P* < 0.05 was used to define significantly enriched pathways.

The crosstalk between pathways was calculated based on a cumulative hypergeometric distribution using the following formula:

(1) $$\begin{array}{c} \;P=1-\overset{x}{\underset{k=0}{\sum }}\frac{\left(\begin{array}{c} m\\ k \end{array}\right)\left(\begin{array}{c} N-m\\ n-k \end{array}\right)}{\left(\begin{array}{c} N\\ n \end{array}\right)}, \end{array}$$

where *N* is the total number of genes in the human genome, *n* is the number of genes in one pathway, *m* is the number of genes in a different pathway, and *x* is the number of genes that are common to both pathways. *P* < 0.05 was defined as the cutoff value for significant crosstalk between pathways.

#### 2.2.3. Gene Ontology Enrichment Analysis

The cumulative hypergeometric distribution was also used for the gene ontology (GO) functional enrichment analysis. Gene sets were mapped to GO terms according to biological process (BP), cellular component (CC), and molecular function (MF). Using the above formula, *N* is the total number of genes in the human genome, *n* is the number of gene sets in one pathway, *m* is the number of genes annotated with a particular GO term, and *x* is the number of genes that show overlap between the pathway and the GO term. The* P* value was adjusted using the Benjamini and Hochberg false discovery rate (FDR) to determine statistical significance and GO terms were selected based on an FDR < 0.05.

#### 2.2.4. Cluster Analysis of Pathways

Significant crosstalk was determined for each pair of pathways based on a hypergeometric test, and corresponding −log 10* P* values formed the crosstalk matrix. Hierarchical clustering was implemented to group pathways into clusters based on the crosstalk matrix. A heatmap was generated to visualize the results using the gplots2 package of R software.

#### 2.2.5. Identification of LAPs

The *k*-clique method in the SubpathwayMiner software was used to mine LAPs based on targets of differentially expressed miRNA as well as mRNA expression data [28, 29]. This method identified LAPs based on the closeness of genes in pathways as measured by the distance parameter *k* such that the distance between any genes in an LAP was <*k*. The default value *k* = 4 was used to identify significantly enriched LAPs.

## 3. Results

### 3.1. miRNAs Affecting Pathways Dysregulated in MG

A total of 1135 differentially expressed mRNAs were obtained from the screen, including 551 upregulated mRNAs and 584 downregulated mRNAs that were enriched in 13 (P_1_) and 8 (P_2_) pathways, respectively. A total of 46 differentially expressed miRNAs were identified, including 21 upregulated miRNAs and 25 downregulated miRNAs, and their predicted targets were enriched in 64 (P_3_) and 68 (P_4_) pathways, respectively (Tables S1, S2, and S3).

**Figure 1:**
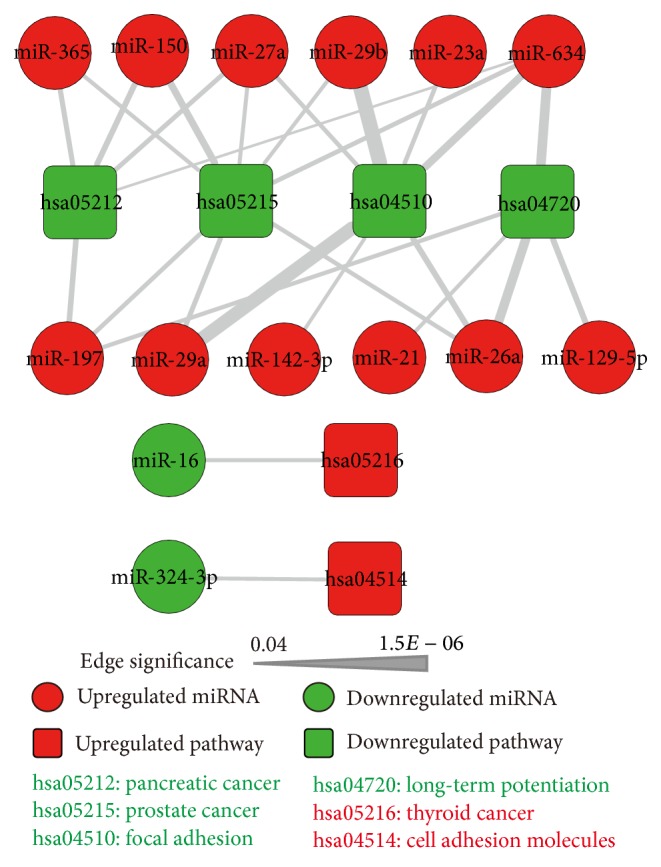
miRNA-regulated pathways in MG. Red and green rectangles represent up- and downregulated pathways, respectively; red and green circles represent up- and downregulated miRNAs, respectively. Lines represent regulatory interactions between miRNAs and their target pathways, with wider lines representing a more significant interaction.

Since miRNAs are negative regulators of mRNAs [30], pathways enriched for differentially expressed mRNAs were used to filter predicted targets based on inverse miRNA-mRNA regulation. The intersections of P_1_ + P_4_ and P_2_ + P_3_ were defined as up- and downregulated pathways, respectively. Two upregulated and four downregulated pathways potentially targeted by two downregulated and 12 upregulated miRNAs were identified (Figure 1). Of the 14 dysregulated miRNAs found in this study, all but miR-634 have been validated in other autoimmune diseases based on the Human MicroRNA Disease Database (HMDD) and miR2Disease databases (Table S4) [31, 32]. For example, miR-21 is upregulated in several autoimmune diseases such as multiple sclerosis [33], psoriasis [34], and systemic lupus erythematosus [35], which shares some etiological features with MG, implying that the miRNAs identified for MG are common features of autoimmune diseases.

A total of 14 dysregulated miRNAs were mapped to chromosomes using miRBase (release 20) [36]. Consistent with the findings of a previous study [37], miRNA genes affecting the same pathway were clustered together. A cluster was defined as a maximum distance of 10 kb between two miRNA genes. We found hsa-miR-29a/29b (mapped to chromosome 7q32.3) regulated the pathway hsa05215 and along with hsa-miR-27a/23a (mapped to chromosome 19p13.13) coregulated hsa04510, suggesting a synergistic interaction.

### 3.2. MG-Related Genes Mediate Pathway Crosstalk

**Figure 2:**
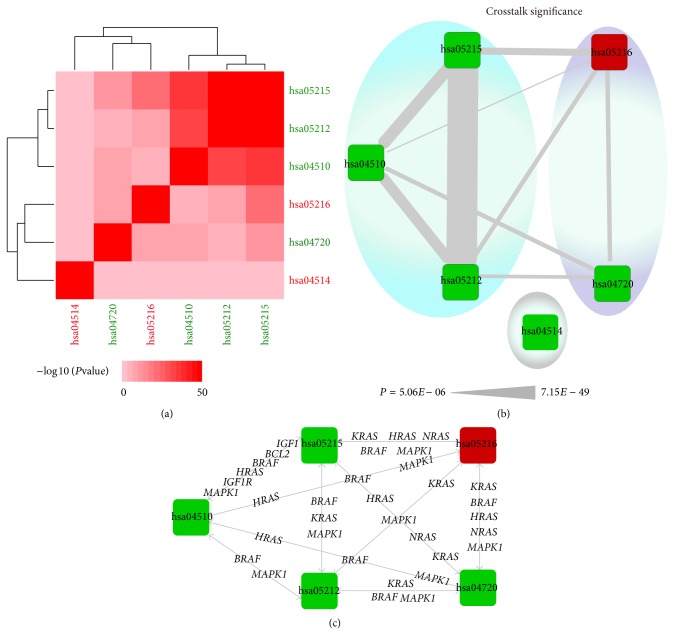
Pathway crosstalk analysis and functional clustering. (a) Hierarchical clustering of dysregulated pathways based on −log 10* P* values of their interactions; stronger interactions are represented by a more intense color. (b) Crosstalk between dysregulated pathways. Rectangular nodes represent pathways and edges denote crosstalk between two pathways, with wider edges representing greater interaction between two pathways. Two clusters (blue and purple) were identified; one pathway did not cluster with the others (grey). (c) Crosstalk between pathways mediated by MG-related genes.

Crosstalk was observed between all dysregulated pathways except hsa04514 (Table S5). Two clusters were identified: one consisting of pathways hsa05215, hsa05212, and hsa04510 and the other of hsa05216 and hsa04720 (Figures 2(a) and 2(b)). Importantly, genes such as* HRAS*,* MAPK1*, and* BRAF* were engaged in crosstalk (Figure 2(c)) and have been previously found to be associated with MG [38, 39]. These results suggest that crosstalk between multiple dysregulated pathways is responsible for the heterogeneity of MG.

It is worth noting that pathway hsa04720 (*synaptic long-term potentiation* (LTP))—which is important for long-lasting increases in synaptic efficacy that serve as the basis for learning and memory—interacted with each of the other four pathways via five immune-related genes, that is,* BRAF*,* MAP2K1* and* MAP2K2*, and* MAPK1* and* MAPK3*. The signaling cascade involving these genes, that is,* BRAF-MEK1/2* (*MAP2K1/2*)-*ERK1*/*2* (*MAPK1/3*), is part of the MAPK signaling pathway, which is involved in cell proliferation, differentiation, and migration [40] and was recently found to be dysregulated in MG [41].

### 3.3. Detecting Key Genes Regulated by miRNAs in Crosstalk Pathways

**Figure 3:**
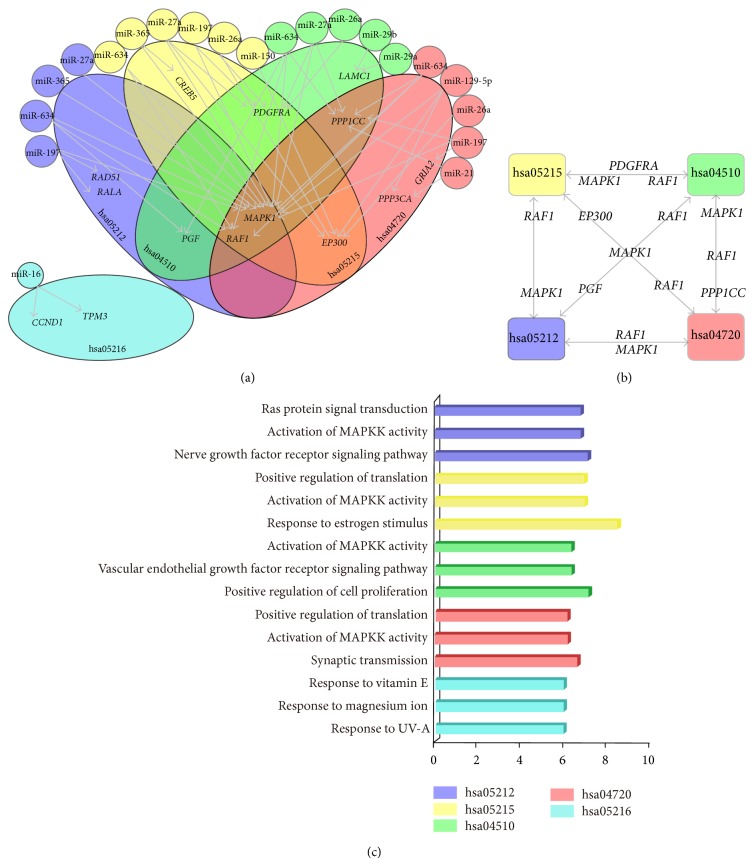
Key genes regulated by miRNAs involved in dysregulated pathways. (a) Key genes regulated by miRNAs. Circles represent miRNAs and large ellipses represent pathways with key genes. miRNAs and their target pathways are represented by the same color; arrows denote key genes regulated by miRNAs. (b) Crosstalk between downregulated pathways through key genes. (c) Top three GO functions of key genes in each pathway; the histogram represents −log 2 (FDR).

miRNAs and their target genes that were enriched in the identified pathways and whose differential expression was inversely related were selected, for a total of 14 key genes in five pathways targeted by 11 miRNAs (Figure 3(a)). Six miRNA-gene pairs were already experimentally verified, including miR-16 and* CCND1* [42], miR-16 and* TPM3* [43], miR-29a/b and* LAMC1* [44], miR-197 and* RAD51* [45], and miR-150 and* EP300* [46]. Moreover, six genes (*MAPK1*,* RAF1*,* PGF*,* PDGFRA*,* EP300*, and* PPP1CC*) were common to more than one MG-related pathway (Figure 3(b)). In particular, four downregulated pathways interacted through* MAPK1* and* RAF1*, reflecting their potential involvement in MG pathogenesis.

Key genes were organized into hierarchical categories by functional enrichment analysis. A total of 133 GO terms, including 84 BP, 35 MF, and 14 CC terms, were obtained (Table S6). Key genes were primarily involved in BP such as biological regulation, response to stimulus, metabolic, single-organism, cell, and developmental processes, establishment of localization, signaling, and cellular component organization; CC such as membrane, cell part, and macromolecular complex; and MF such as catalytic activity and binding.

### 3.4. Key Genes Enriched in LAPs

Recent studies have suggested that pathways are not globally disrupted, but rather only in a local area [47, 48]; thus, key genes enriched in 36 LAPs were considered as being important for MG (Table S7). Though, of the MG-related crosstalk pathways, three were cancer related, some key genes were enriched in immune-related LAPs within these pathways, which could explain the association between MG and cancer-related pathways (Figures S1, S2, and S4). Key genes and their regulatory miRNAs were mapped to specific pathways and LAPs, and their contributions to the molecular mechanism of MG are discussed below.

**Figure 4:**
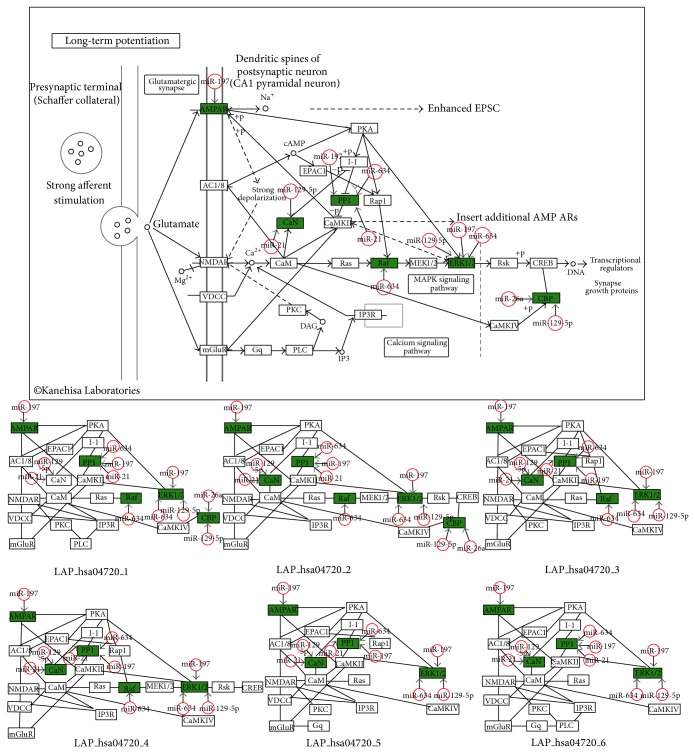
Representative illustration of key gene distribution (hsa04720 and LAP_hsa04720s). Downregulated key genes are shown in green; red open circles represent their regulatory miRNAs. LAP_hsa04720s and associated key genes are shown below the hsa04720 pathway.

*Synaptic LTP (hsa04720)*. This pathway is implicated in various neurological disorders, including Parkinson's disease [49], stroke [50], multiple sclerosis [51], and Alzheimer's disease [52]. Six downregulated hsa04720-related genes (*RAF1*,* EP300*,* PPP1CC*,* MAPK1*,* GRIA2*, and* PPP3CA*) were predicted to be targeted by five upregulated miRNAs (hsa-miR-634, hsa-miR-26a, hsa-miR-129-5p, hsa-miR-197, and hsa-miR-21). The structures of the six LAPs were similar, indicating that genes in the whole pathway and LAPs were tightly linked (Figure 4). All six genes have been directly or indirectly implicated in the regulation of LTP; thus, their dysregulation by miRNAs would be expected to result in neurological diseases.

*Prostate Cancer (hsa05215)*. Five downregulated genes (*EP300*,* RAF1*,* PDGFRA*,* CREB5*, and* MAPK1*) were predicted to be targeted by six miRNAs (hsa-miR-150, hsa-miR-634, hsa-miR-197, hsa-miR-26a, hsa-miR-365, and hsa-miR-27a). The regulation of* EP300* by miR-150 has been validated [46], and miR-150 is overexpressed in human lymphocyte cells and lymphocyte-derived exosomes [53]. MiR-150 is involved in immune regulation, including B and T cell differentiation and NK cell development [54, 55]; therefore, miR-150 may activate T cell immune responses by targeting* EP300*.* CREB5* has been implicated in Alzheimer's and Parkinson's diseases [56]. Here it was predicted that the downregulation of* CREB5* was due to regulation by miR-365; this was supported by a recent study showing reduced* CREB* activity in an experimental MG model [57]. Key genes were also enriched in immune-related LAPs that are potentially related to MG; for example,* PDGFRA*,* MAPK1*, and* RAF1* were enriched in LAP_05215_7 and LAP_05215_1, both of which are involved in cytokine-cytokine receptor interactions and MAPK signaling, which play important roles in MG pathogenesis (Figure S1) [41].

*Pancreatic Cancer (hsa05212)*. Five downregulated genes (*RALA*,* MAPK1*,* RAF1*,* RAD51*, and* PGF*) were predicted to be targeted by four miRNAs (hsa-miR-634, hsa-miR-197, hsa-miR-365, and hsa-miR-27a). LAP_05212_10 comprised* RAD51* and* BRCA2*. The regulation of* RAD51* by miR-197 has been experimentally verified [45]. Four of the genes were enriched in immune-related LAPs. For example,* MAPK1*,* RAF1*, and* RALA* were enriched in LAP_05212_3, LAP_05212_4, and LAP_05212_5, which is linked to the PI3K-Akt and MAPK signaling pathways.* PGF* was enriched in LAP_05212_1, which is involved in ErbB and Jak-STAT signaling pathways (Figure S2).

*Focal Adhesion (hsa04510)*. This pathway has been implicated in nervous system disorders such as depression in genomewide molecular pathway analyses [58, 59]. Six downregulated genes (*LAMC1*,* RAF1*,* PDGFRA*,* PPP1CC*,* PGF*, and* MAPK1*) were predicted to be targeted by five miRNAs (hsa-miR-29b, hsa-miR-29a, hsa-miR-634, hsa-miR-26a, and hsa-miR-27a). The regulation of* LAMC1* by miR-29a/b has been validated [44]. These genes were enriched in immune-related LAPs that are linked to cytokine-cytokine receptor interaction pathway, MAPK signaling pathway, and PI3K-Akt signaling pathway (Figure S3).

*Thyroid Cancer (hsa05216)*. This pathway was upregulated in MG and is potentially regulated by miR-16 and its predicted targets* CCND1* and* TPM3*. MiR-16 is downregulated in peripheral blood mononuclear cells and sera of MG patients [6, 8]. The regulatory interaction between miR-16 and* CCND1* and* TPM3* has been experimentally verified in humans [42, 43].* CCND1*—a downstream gene in LAP_hsa05216_4—is a cell cycle-related gene that controls the G1/S transition and is a target of miR-16 [42].* TPM3*—an upstream gene in LAP_hsa05216_2—is an actin-binding protein that mediates myosin-actin interaction and stabilizes microfilaments in muscle [60]. Thus, the upregulation of these two genes by miR-16 dysregulation is likely to be involved in MG (Figure S4).

## 4. Discussion

MG is an autoimmune disease resulting from the failure of neuromuscular transmission due to binding of autoantibodies to proteins involved in signaling at the neuromuscular junction (NMJ). Available treatments include acetylcholinesterase drugs, immunomodulatory agents, plasma exchange, intravenous immunoglobulin administration, and thymectomy. However, some MG patients are unresponsive to conventional therapies or suffer adverse reactions from long-term use of immunomodulatory drugs such as steroids or immunosuppressants [61]. Accordingly, there is a need for treatments that target specific factors involved in MG.

The present study identified six dysregulated pathways potentially targeted by 14 miRNAs. The LTP pathway is associated with multiple neurological disorders [49–52] and was found here to be involved in MG. The NMJ connects neurons to target muscles, and this communication is similar to that between two neurons in LTP. With the exception of miR-634, all miRNAs associated with pathways dysregulated in MG were autoimmune disease-related ones. For instance, miR-23a expression is upregulated in Crohn's disease [62] and lupus nephritis [63] in a pattern similar to that observed in MG. miRNAs dysregulated in MG have known functions as activators of the T cell phenotype and promote T cell-driven B cell differentiation into immunoglobulin-secreting plasma cells [35]; given that MG is a T cell-regulated disorder [64], it can be speculated that miRNA dysfunction in MG can activate T cell-induced immune responses and stimulate the synthesis of autoantibodies by B cells.

Essential genes are often implicated in multiple pathways, which can interact to give rise to a disease state [65]. For example, in MG, NF-*κ*B signaling regulates immune responses [13], and the activation of this pathway depends on PI3K-Akt signaling [66]. In the present study, significant crosstalk was detected between pathways dysregulated in MG, which likely accounts for the heterogeneity of the disease. Notably, members of the* Ras* gene family were implicated in this crosstalk; it has been previously reported that some* Ras* gene family members are linked to the acceleration of clinical features of MG [38].

A set of key genes regulated by miRNAs associated with pathways dysregulated in MG was identified; six of these miRNA-gene pairs were already experimentally validated. For example,* LAMC1* in the “*focal adhesion*” pathway has been implicated in processes such as cell adhesion, differentiation, migration, signaling, neurite outgrowth, and metastasis; miR-29a/b regulates this pathway and inhibits cancer cell migration and invasion by targeting* LAMC1* [44]. The key genes that were identified mediated crosstalk between the dysregulated pathways and included* MAPK1* and* RAF1*, two factors in MAPK signaling that play important roles in the immune response [39], neurotransmission [67], and neurological diseases such as Alzheimer's disease and Parkinson's disease as well as amyotrophic lateral sclerosis [68]. Thus, the pathogenesis of MG likely involves these key genes and their dysregulation by aberrantly expressed miRNAs, which affects multiple signaling pathways.

LAPs enriched in key genes were also examined to obtain more detailed information on their specific roles in each pathway. The genes were enriched in immune-related LAPs. For instance, previous studies have indicated that cytokines mediate immune responses in MG [69]; it was found here that* PDGFRA*—an upstream key gene in LAP_04510_13 and LAP_05215_7 whose product binds another key gene product,* PGF*—triggers cytokine-cytokine receptor interactions.

## 5. Conclusions

In this study, pathway enrichment, pathway crosstalk, functional enrichment, and LAP analyses were combined to elucidate miRNA-gene interactions in the context of pathways relevant to MG. Crosstalk between pathways dysregulated in MG was detected, and key genes in these pathways were identified that are likely involved in MG pathogenesis, providing potential new targets for therapeutic interventions.

## Supplementary Material

Table S1: MiRNAs and mRNAs differentially expressed in MG Table S1 lists the miRNAs and mRNAs differentially expressed in MG. A total of 46 differentially expressed miRNAs were identified, including 21 upregulated miRNAs and 25 downregulated miRNAs. A total of 1135 differentially expressed mRNAs were obtained, including 551 upregulated mRNAs and 584 downregulated mRNAs.
Table S2: The predicted targets of differentially expressed miRNAs
Table S2 lists the predicted targets of differentially expressed miRNAs. A total of 46 differentially expressed miRNAs, 9567 predicted targets, 34963 miRNA-target regulations were obtained.
Table S3: Pathways enriched in differentially expressed mRNAs and predicted target genes of differentially expressed miRNAs
Table S3 presents significant pathways enriched by differentially expressed mRNAs and predicted genes of differentially expressed miRNAs. The up- and down-regulated mRNAs were significantly enriched in 13 pathways (define as P_1_) and 8 pathways (define as P_2_), respectively. The predicted targets of up- and down-regulated miRNAs were significantly enriched in 64 pathways (define as P_3_) and 68 pathways (define as P_4_), respectively.
