# Corrigendum to “Detecting Key Genes Regulated by miRNAs in Dysfunctional Crosstalk Pathway of Myasthenia Gravis”

**DOI:** 10.1155/2017/9281250

**Published:** 2017-08-14

**Authors:** Yuze Cao, Jianjian Wang, Huixue Zhang, Qinghua Tian, Lixia Chen, Shangwei Ning, Peifang Liu, Xuesong Sun, Xiaoyu Lu, Chang Song, Shuai Zhang, Bo Xiao, Lihua Wang

**Affiliations:** ^1^Department of Neurology, The Second Affiliated Hospital, Harbin Medical University, Harbin, Heilongjiang 150081, China; ^2^Department of Neurology, Xiangya Hospital, Central South University, Changsha, Hunan 410008, China; ^3^College of Bioinformatics Science and Technology, Harbin Medical University, Harbin, Heilongjiang 150081, China

In the article titled “Detecting Key Genes Regulated by miRNAs in Dysfunctional Crosstalk Pathway of Myasthenia Gravis” [1], it was not clearly stated how to identify dysfunctional pathways in myasthenia gravis (MG), as raised by Dr. Panse in [2]. The authors clarified how to overcome the issue in their response [3]. Accordingly, the Results and Discussion sections are corrected as follows.

In the Results section, “Since miRNAs are negative regulators of mRNAs, pathways enriched for differentially expressed mRNAs were used to filter predicted targets based on inverse miRNA-mRNA regulation. The intersections of P_1_ + P_4_ and P_2_ + P_3_ were defined as up- and downregulated pathways, respectively” should be corrected to “Since miRNAs are negative regulators of mRNAs, pathways enriched for differentially expressed mRNAs were used to filter predicted targets based on inverse miRNA-mRNA regulation. In brief, we applied pathway filter analyses instead of using miRNA-mRNA regulatory pairs directly. The intersections of P_1_ + P_4_ and P_2_ + P_3_ were defined as up- and downregulated pathways, respectively.”

In the Discussion section, “However, some MG patients are unresponsive to conventional therapies or suffer adverse reactions from long-term use of immunomodulatory drugs such as steroids or immunosuppressants” should be corrected to “However, some MG patients are unresponsive to conventional therapies or suffer adverse reactions from long-term use of immunomodulatory drugs such as steroids or immunosuppressants. The category of MG patients is complex, which could be classified according to the antibody specificity, age at onset, type of course, and thymus histology. Therefore, we do not consider anti-AChR-positive and anti-AChR-negative patients specifically.”

## References

[1] Cao Y., Wang J., Zhang H. (2015). Detecting key genes regulated by miRNAs in dysfunctional crosstalk pathway of myasthenia gravis. *BioMed Research International*.

[2] Le Panse R. (2017). Comment on ‘detecting key genes regulated by miRNAs in dysfunctional crosstalk pathway of myasthenia gravis’. *BioMed Research International*.

[3] Cao Y., Wang J., Zhang H. (2017). Response to: comment on ‘detecting key genes regulated by miRNAs in dysfunctional crosstalk pathway of myasthenia gravis’. *BioMed Research International*.

