# Supplementary material for: Detecting Key Genes Regulated by miRNAs in Dysfunctional Crosstalk Pathway of Myasthenia Gravis
Source: Biomed Res Int. 2015 Feb 1;2015:724715. doi: 10.1155/2015/724715 (PMC4331476; doi:10.1155/2015/724715)
Supplement: Supplementary file 1 — Table S1: MiRNAs and mRNAs differentially expressed in MG Table S1 lists the miRNAs and mRNAs differentially expressed in MG. A total of 46 differentially expressed miRNAs were identified, including 21 upregulated miRNAs and 25 downregulated miRNAs. A total of 1135 differentially expressed mRNAs were obtained, including 551 upregulated mRNAs and 584 downregulated mRNAs. Table S2: The predicted targets of differentially expressed miRNAs Table S2 lists the predicted targets of differentially expressed miRNAs. A total of 46 differentially expressed miRNAs, 9567 predicted targets, 34963 miRNA-target regulations were obtained. Table S3: Pathways enriched in differentially expressed mRNAs and predicted target genes of differentially expressed miRNAs Table S3 presents significant pathways enriched by differentially expressed mRNAs and predicted genes of differentially expressed miRNAs. The up- and down-regulated mRNAs were significantly enriched in 13 pathways (define as P1) and 8 pathways (define as P2), respectively. The predicted targets of up- and down-regulated miRNAs were significantly enriched in 64 pathways (define as P3) and 68 pathways (define as P4), respectively. [file 724715.f1.zip › Optional Supplementary Materials/Optional Supplementary Materials.docx]

Detecting key genes regulated by miRNAs in dysfunctional crosstalk pathway of Myasthenia Gravis

Yuze Cao, ‡^1, 2^ Jianjian Wang, ‡^1^ Huixue Zhang,^1^ Qinghua Tian,^1^ Lixia Chen,^1^ Shangwei Ning,^3^ Peifang Liu,^1^ Xuesong Sun,^1^ Xiaoyu Lu,^1^ Chang Song,^1^ Shuai Zhang,^1^ Bo Xiao,*^2^ and Lihua Wang*^1^

^1^Department of Neurology, The Second Affiliated Hospital, Harbin Medical University, Harbin 150081, Heilongjiang Province, China.

^2^Departtment of Neruology, Xiangya Hospital, Central Sounth University,Changsha 410008, Hunan Provience, China.

^3^College of Bioinformatics Science and Technology, Harbin Medical University, Harbin 150081, Heilongjiang Province, China.

*Correspondence should be addressed to Lihua Wang; wanglh211@163.com and Bo Xiao; xiaobo_xy@126.com.

‡The first two authors contributed equally to this work.

The supplementary information contains the seven tables and four figures: Table S1 lists the miRNAs and mRNAs differentially expressed in MG. Table S2 lists the predicted targets of differentially expressed miRNAs. Table S3 presents significant pathways enriched by differentially expressed mRNAs and predicted genes of differentially expressed miRNAs. Table S4 shows the associations between miRNAs dysregulated in MG and other autoimmune diseases based on HMDD and miR2Disease databases. Table S5 presents the interaction between MG dysfunctional pathways. Table S6 lists enriched GO terms of key genes and their functional categories. Table S7 shows the key genes enriched in LAPs that were considered as being important for MG. Table S1-3, S6 and S7 were submitted as excel formats. Figures S1-S4 present key genes and their regulatory miRNAs in the specific pathways and LAPs. We also provide the raw data of expression profiles for MG as zip formats, which comprised 30 samples from 20 MG patients and 10 control subjects, for a total of 12,814 genes (accession number: E-MEXP-518).

**Supplementary Tables**

**Table S1.** MiRNAs and mRNAs differentially expressed in MG

**Table S2.** The predicted targets of differentially expressed miRNAs

**Table S3.** Pathways enriched in differentially expressed mRNAs and predicted target genes of differentially expressed miRNAs

**Table S4.** Associations between miRNAs dysregulated in MG and other autoimmune diseases based on HMDD and miR2Disease databases

| miRNA name | Disease name | PMID | Description |
| --- | --- | --- | --- |
| hsa-mir-150 | Psoriasis | 21687694 | differentially expressed |
| hsa-mir-197 | Psoriasis | 21687694 | differentially expressed |
| hsa-mir-197 | Diabetes Mellitus, Type 2 | 20651284 | decreased in plasma |
| hsa-mir-197 | Psoriasis | 21687694 | differentially expressed |
| hsa-mir-365 | Lupus Vulgaris | 18998140 | miR-365: downregulation |
| hsa-mir-365 | Lupus Vulgaris | 18998140 | miR-365: downregulation |
| hsa-mir-27a | Diabetes Mellitus, Type 1 | 24279768 | hsa-miR-27a were shared among T1D, T2D |
| hsa-mir-29a | Diabetic Nephropathies | 22211842 | TGF-beta1 activates Smad3 to regulate microRNAs that mediate renal fibrosis. Of them, miR-21 and miR-192 are upregulated but miR-29 and miR-200 families are downregulated. |
| hsa-mir-29a | Multiple Sclerosis | 22772450 | miR-29ab Deficiency Identifies a Negative Feedback Loop Controlling Th1 Bias That Is Dysregulated in Multiple Sclerosis. |
| hsa-mir-29a | Scleroderma, Localized | 21129921 | Circulating miR-29a levels in patients with scleroderma spectrum disorder |
| hsa-mir-29a | Scleroderma, Systemic | 21129921 | Circulating miR-29a levels in patients with scleroderma spectrum disorder |
| hsa-mir-26a | ulcerative colitis (UC) | 18835392 | MicroRNAs are differentially expressed in ulcerative colitis and alter expression of macrophage inflammatory peptide-2 alpha. |
| hsa-mir-29b | Diabetic Nephropathies | 22211842 | TGF-beta1 activates Smad3 to regulate microRNAs that mediate renal fibrosis. Of them, miR-21 and miR-192 are upregulated but miR-29 and miR-200 families are downregulated. |
| hsa-mir-29b | Diabetic Nephropathies | 22211842 | TGF-beta1 activates Smad3 to regulate microRNAs that mediate renal fibrosis. Of them, miR-21 and miR-192 are upregulated but miR-29 and miR-200 families are downregulated. |
| hsa-mir-29b | Multiple Sclerosis | 22772450 | miR-29ab Deficiency Identifies a Negative Feedback Loop Controlling Th1 Bias That Is Dysregulated in Multiple Sclerosis. |
| hsa-mir-29b | Multiple Sclerosis | 22772450 | miR-29ab Deficiency Identifies a Negative Feedback Loop Controlling Th1 Bias That Is Dysregulated in Multiple Sclerosis. |
| hsa-mir-29b | Scleroderma, Systemic | 22307526 | The expression of the miRNA was correlated with Systemic Scleroderma fibrosis |
| hsa-mir-29b | Scleroderma, Systemic | 22307526 | The expression of the miRNA was correlated with Systemic Scleroderma fibrosis |
| hsa-mir-23a | Crohn Disease | 22386737 | The miRNA showed significantly higher levels in the blood from patients with CD compared with the healthy controls. |
| hsa-mir-23a | Lupus Vulgaris | 18998140 | miR-23a: upregulation |
| hsa-miR-142-3p | Lupus Erythematosus, Systemic | 22549634 | Decreased miR-142-3p/5p expression causes CD4(+) T cell activation and B cell hyperstimulation in systemic lupus erythematosus. |
| hsa-miR-142-3p | Psoriasis | 21807764 | miR-142-3p: deregulated |
| hsa-miR-142-3p | Scleroderma, Systemic | 21883400 | Serum miR-142-3p levels in patients with SSc (systemic sclerosis) were significantly higher than in patients with SSD (scleroderma spectrum disorder), SLE (systemic lupus erythematosus) or DM (dermatomyositis), and healthy control groups. Patients with increased miR-142-3p levels tended to have a short sublingual frenulum. |
| hsa-mir-129-5p | Scleroderma, Systemic | 22403442 | IL-17A signaling, not IL-17F, has an antifibrogenic effect via the upregulation of miR-129-5p and the downregulation of connective tissue growth factor and α1(I) collagen. |
| hsa-mir-129-5p | Scleroderma, Systemic | 22403442 | IL-17A signaling, not IL-17F, has an antifibrogenic effect via the upregulation of miR-129-5p and the downregulation of connective tissue growth factor and α1(I) collagen. |
| hsa-mir-21 | Colitis, Ulcerative | 20586854 | miR-21:Upregulated miRNA may be responsible for the development of intestinal inflammation in UC |
| hsa-mir-21 | Demyelinating Diseases | 22517757 | Both in vivo and in vitro, myelin antigen stimulation resulted in significant up-regulation of miR-301a, miR-21, and miR-155. |
| hsa-mir-21 | Dermatitis, Atopic | 17622355 | miR-21 was significantly up-regulated both psoriasis (p<0.001) and atopic eczema (p<0.001) as compared with healthy skin. |
| hsa-mir-21 | Diabetes Mellitus | 21613227 | microRNA-21 orchestrates high glucose-induced signals to TORC1 for renal cell pathology in diabetes. |
| hsa-mir-21 | Diabetes Mellitus, Type 2 | 20651284 | decreased in plasma |
| hsa-mir-21 | Diabetic Nephropathies | 22211842 | TGF-beta1 activates Smad3 to regulate microRNAs that mediate renal fibrosis. Of them, miR-21 and miR-192 are upregulated but miR-29 and miR-200 families are downregulated. |
| hsa-mir-21 | Inflammatory Bowel Diseases | 23224068 | PDCD4/miR-21 dysregulation in inflammatory bowel disease-associated carcinogenesis |
| hsa-mir-21 | Inflammatory Bowel Diseases | 23288924 | Over expression of mir-RNAs-155 and 21 targeting mismatch repair proteins in Inflammatory Bowel Diseases |
| hsa-mir-21 | Lupus Erythematosus, Systemic | 21602271 | miR-21 regulates aberrant T cell responses through regulation of PDCD4 expression.Upregulated miR-21 affects PDCD4 expression and regulates aberrant T cell responses in human SLE. |
| hsa-mir-21 | Multiple Sclerosis | 21875645 | In peripheral mononuclear cells, a statistically significant increased expression of miR-21, miR-146a and -b was observed in relapsing remitting (RR)MS patients as compared with controls. |
| hsa-mir-21 | Psoriasis | 17622355 | miR-21 was significantly up-regulated both psoriasis (p<0.001) and atopic eczema (p<0.001) as compared with healthy skin. |
| hsa-mir-21 | Psoriasis | 21807764 | deregulated |
| hsa-mir-21 | Psoriasis | 22417311 | MiR-21 is up-regulated in psoriasis and suppresses T cell apoptosis. |
| hsa-mir-21 | Scleroderma, Systemic | 22307526 | The expression of the miRNA was correlated with Systemic Scleroderma fibrosis |
| hsa-miR-324-3p | Lupus Vulgaris | 18998140 | miR-324-3p: downregulation |
| hsa-mir-16 | Arthritis, Rheumatoid | 22100329 | deregulated |
| hsa-mir-16 | Arthritis, Rheumatoid | 22100329 | deregulated |
| hsa-mir-16 | Autoimmune Diseases | 17351108 | New Zealand black (NZB) mice (NZB) tissue sourcesof RNA showed a decrease in miR-16 in the spleen; however, theNZB kidney was not decreased in miR-16 expression compared withthe control strain expression. In addition, the NZB-derivedmalignant B-cell line, LNC, had an even greater decreased expressionof miR-16 compared with C57Bl6 spleen. |
| hsa-mir-16 | Autoimmune Diseases | 17351108 | New Zealand black (NZB) mice (NZB) tissue sourcesof RNA showed a decrease in miR-16 in the spleen; however, theNZB kidney was not decreased in miR-16 expression compared withthe control strain expression. In addition, the NZB-derivedmalignant B-cell line, LNC, had an even greater decreased expressionof miR-16 compared with C57Bl6 spleen. |
| hsa-mir-16 | Crohn Disease | 22386737 | The miRNA showed significantly higher levels in the blood from patients with CD compared with the healthy controls. |
| hsa-mir-16 | Crohn Disease | 22386737 | The miRNA showed significantly higher levels in the blood from patients with CD compared with the healthy controls. |

**Table S5.** Crosstalk between pathways dysregulated in MG

| pathway name | pathway name | P value |
| --- | --- | --- |
| hsa05212 | hsa05215 | 7.15E-49 |
| hsa04510 | hsa05215 | 4.40E-34 |
| hsa04510 | hsa05212 | 2.23E-31 |
| hsa05215 | hsa05216 | 1.89E-20 |
| hsa04720 | hsa05215 | 3.68E-11 |
| hsa05216 | hsa04720 | 1.34E-09 |
| hsa05216 | hsa05212 | 1.51E-09 |
| hsa04720 | hsa04510 | 1.67E-09 |
| hsa04720 | hsa05212 | 2.20E-06 |
| hsa05216 | hsa04510 | 5.06E-06 |
| hsa04510 | hsa04514 | 0.1505791 |
| hsa05216 | hsa04514 | 0.5069586 |
| hsa05212 | hsa04514 | 1 |
| hsa05215 | hsa04514 | 1 |
| hsa04720 | hsa04514 | 1 |

**Table S6.** Enriched GO terms of key genes and their functional categories

**Table S7.** Key genes enriched in LAPs

**Supplementary Figure****s**





**Figure S1.** Representative illustration of key gene distribution (hsa05215 and LAP_hsa05215s). Downregulated key genes are shown in green; red open circles represent their regulatory miRNAs and solid circles represent experimentally verified interactions between miRNAs and their targets. LAP_hsa05215s and associated key genes are shown below the hsa05215 pathway.





**Figure S2.** Representative illustration of key gene distribution (hsa05212 and LAP_hsa05212s). Downregulated key genes are shown in green; red open circles represent their regulatory miRNAs and solid circles represent experimentally verified interactions between miRNAs and their targets. LAP_hsa05212s and associated key genes are shown below the hsa05212 pathway.





**Figure S3.** Representative illustration of key gene distribution (hsa04510 and LAP_hsa04510s). Downregulated key genes are shown in green; red open circles represent their regulatory miRNAs and solid circles represent experimentally verified interactions between miRNAs and their targets. LAP_hsa04510s and associated key genes are shown below the hsa04510 pathway.


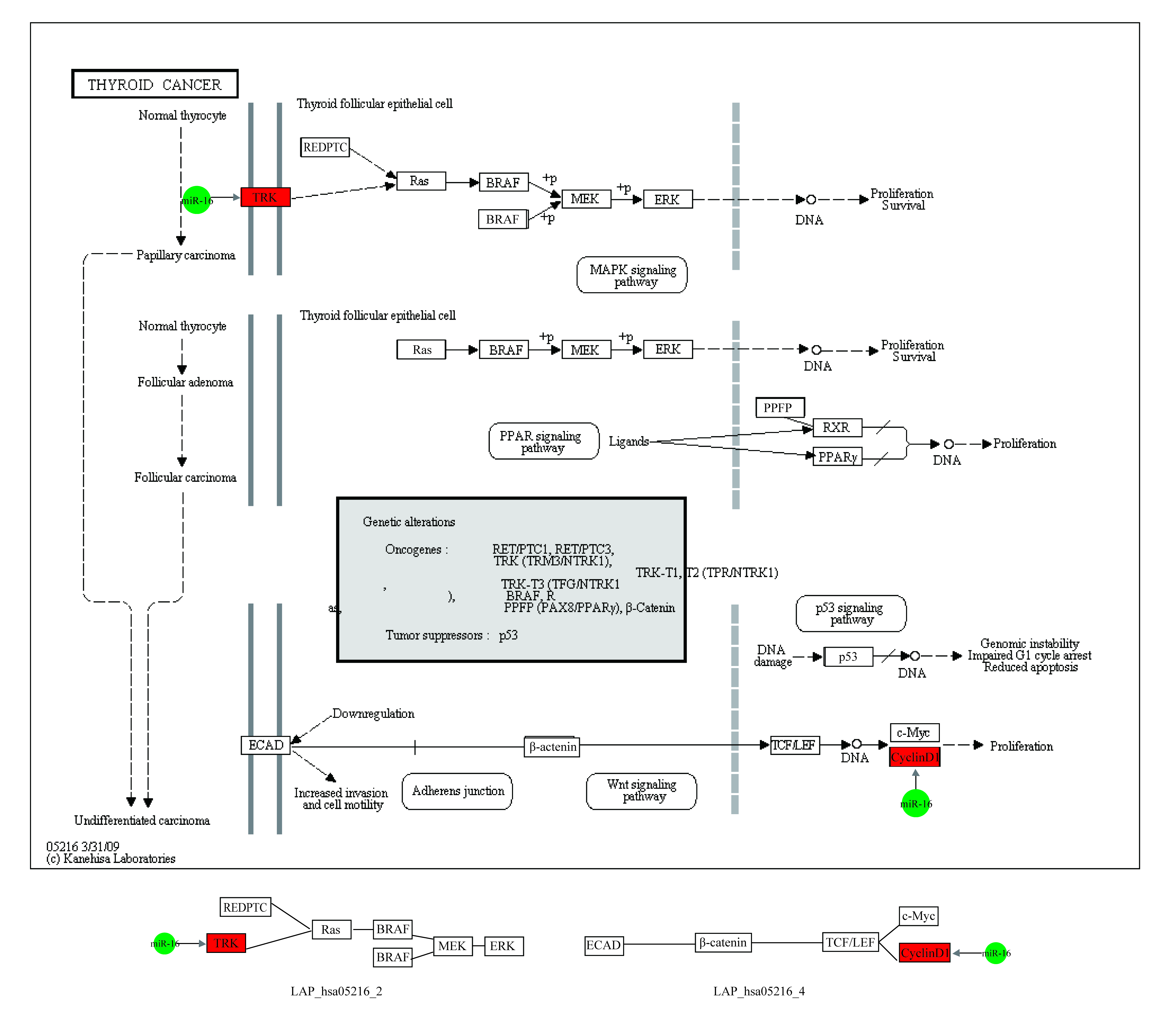


**Figure S4.** Representative illustration of key gene distribution (hsa05216 and LAP_hsa05216s). Upregulated key genes are shown in red; green solid circles represent experimentally verified interactions between miRNAs and their targets. LAP_hsa05216s and associated key genes are shown below the hsa04510 pathway.
